# Dual Action of Dipyridothiazine and Quinobenzothiazine Derivatives—Anticancer and Cholinesterase-Inhibiting Activity

**DOI:** 10.3390/molecules25112604

**Published:** 2020-06-03

**Authors:** Jakub Jończyk, Justyna Godyń, Ewelina Stawarska, Beata Morak-Młodawska, Małgorzata Jeleń, Krystian Pluta, Barbara Malawska

**Affiliations:** 1 Department of Physicochemical Drug Analysis, Faculty of Pharmacy, Jagiellonian University Medical College, Medyczna 9, 30-688 Kraków, Poland; jakub.jonczyk@uj.edu.pl (J.J.); justyna.godyn@uj.edu.pl (J.G.); ewelina.stawarska@student.uj.edu.pl (E.S.); 2 Department of Organic Chemistry, Faculty of Pharmaceutical Sciences in Sosnowiec, The Medical University of Silesia in Katowice Jagiellońska 4, 41-200 Sosnowiec, Poland; bmlodawska@sum.edu.pl (B.M.-M.); manowak@sum.edu.pl (M.J.); pluta@sum.edu.pl (K.P.)

**Keywords:** cholinesterase inhibitors, virtual screening, Alzheimer’s disease, anticancer agents, dipyridothiazines, quinobenzothiazine

## Abstract

The inverse correlation observed between Alzheimer’s disease (AD) and cancer has prompted us to look for cholinesterase-inhibiting activity in phenothiazine derivatives that possess anticancer properties. With the use of in silico and in vitro screening methods, our study found a new biological activity in anticancer polycyclic, tricyclic, and tetracyclic compounds. The virtual screening of a library of 120 ligands, which are the derivatives of azaphenothiazine, led to the identification of 25 compounds that can act as potential inhibitors of acetylcholinesterase (AChE) and butyrylcholinesterase (BuChE). Biological assays revealed the presence of selective inhibitors of *ee*AChE (*electric eel* AChE) or *eq*BuChE (*equine serum* BuChE) and nonselective inhibitors of both enzymes among the tested compounds. Their potencies against *ee*AChE were in a submicromolar-to-micromolar range with IC_50_ values from 0.78 to 19.32 μM, while their IC_50_ values against *eq*BuChE ranged from 0.46 to 10.38 μM. The most potent among the compounds tested was the tetracyclic derivative, 6-(4-diethylaminobut-2-ynyl)-9-methylthioquinobenzothiazine **24**, which was capable of inhibiting both enzymes. 9-Fluoro-6-(1-piperidylethyl)quinobenzothiazine **23** was found to act as a selective inhibitor of *eq*BuChE with an IC_50_ value of 0.46 μM. Compounds with such a dual antitumor and cholinesterase-inhibitory activity can be considered as a valuable combination for the treatment of both cancer and AD prevention. The results presented in this study might open new directions of research on the group of tricyclic phenothiazine derivatives.

## 1. Introduction

Alzheimer’s disease (AD) and cancer are widespread illnesses responsible for a number of deaths and, hence, are considered not only as a medical but also as a social and economic problem in the modern world. The observational epidemiological data revealed an inverse correlation between the two diseases [1,2,3], which led to the initiation of various studies aiming to analyze the background of this association [4,5,6,7]. It has been found that compared to people without Alzheimer’s dementia the risk of developing cancer is lower among patients with AD while the risk of developing AD is lower in patients with a history of cancer [8,9,10]. The results of the current research indicate the link between the molecular mechanisms involved in various types of cancers and AD, and encourage the use of some anticancer agents in the treatment of AD [11,12,13,14]. The knowledge and understanding gained from these studies can be applied for developing new therapies and designing new effective drugs for the treatment of both diseases.

AD is a progressive, complex neurodegenerative disorder that results in memory loss and affects the cognitive functions of the brain. According to the oldest pathophysiological hypothesis, memory impairments associated with AD are caused by the degradation and loss of cholinergic neurons in the central nervous system (CNS) and the impairment of cholinergic neurotransmission [15]. Acetylcholinesterase (AChE, E.C.3.1.1.7) and butyrylcholinesterase (BuChE, E.C.3.1.1.8) are enzymes belonging to the group of serine hydrolases. These enzymes can hydrolyze the acetylcholine (ACh) neurotransmitter and some other choline esters, but to a different extent. The major role of AChE is to catalyze the hydrolysis of ACh in cholinergic synapses, whereas the function of BuChE is less clearly defined and it is thought to play a secondary role [16]. In addition, cholinesterases have been shown to perform some noncholinergic functions. Both AChE and BuChE regulate neuronal development and participate in cellular proliferation, differentiation, and adhesion by modulating the activity of other proteins [17,18,19].

Increased attention has been paid to the function of AChE in CNS due to its involvement in the pathogenesis of AD, which is the most common form of dementia. During AD, the concentration of AChE significantly decreases. On the other hand, the level of BuChE increases, especially in the hippocampus and temporal cortex of the brain which are the regions responsible for cognition and behavior, and compensates for the loss of AChE [20]. Both these enzymes have also been shown to be associated with the formation of β-amyloid (Aβ) fibrils and neurofibrillary tangles (NFTs), the two major hallmarks of AD, as well as with the development of neuroinflammation [21]. The cholinergic hypothesis first led to the introduction of cholinesterase inhibitors such as rivastigmine, donepezil, and galantamine in AD therapy. Since then, many structurally diverse inhibitors of cholinesterase have been developed, as well as the multifunctional ligands—the cholinesterase inhibitors with additional properties that have the potential to act as anti-AD agents. Therefore, AChE and BuChE are still considered as valuable targets in the search for new anti-AD agents.

**Figure 1:**
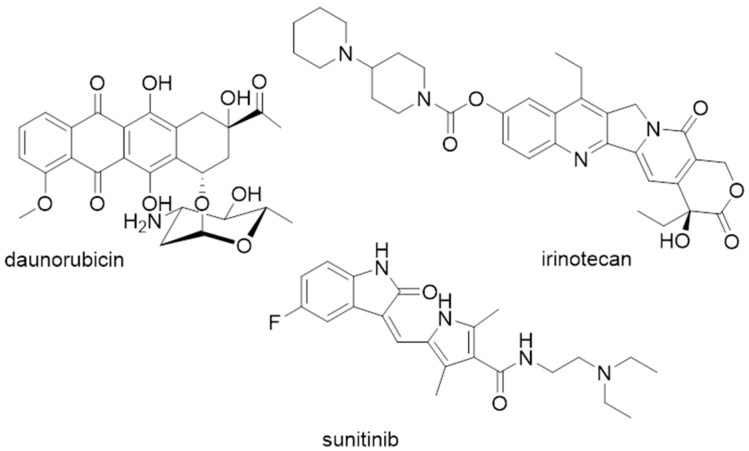
Anticancer drugs with acetylcholinesterase activity.

On the other hand, the involvement of AChE in nonneuronal functions such as the regulation of cell proliferation, differentiation, and apoptosis has suggested that it might play a role in different types of cancers [19,22]. AChE has been found to exhibit a proapoptotic function that is independent of its cholinergic role and is therefore believed to be involved in both tumorigenesis and neurodegenerative diseases [23]. These indicate that AChE might serve as a potential therapeutic target in cancer therapy [24]. Some anticancer drugs, such as doxorubicin, daunorubicin, irinotecan [25], sunitinib [26], and Ru(II) complexes with derivatives of phenothiazine, have been shown to possess AChE-inhibitory effects (Figure 1). Moreover, the cholinesterase-inhibitory potency is identified among different chemical classes of anticancer compounds [27,28]. It is interesting to note that anti-AD drugs including donepezil and galantamine also possess anticancer properties [29].

In the present work, we focus on multifunctional ligands as potential anti AD agents among the group of tricyclic and tetracyclic derivatives endowed with anticancer activity. 

The derivatives of tricyclic dipyridothiazine and tetracyclic quinobenzothiazine displayed varied levels of anticancer activity depending on the type of polycyclic rings and the nature of the substituents [30,31,32,33,34,35,36]. They exhibited cytotoxic as well as antiproliferative action against human peripheral blood mononuclear cells stimulated with phytohemagglutinin A and suppressed the lipopolysaccharide-induced production of tumor necrosis factor-alpha by human whole-blood cell cultures. In addition, the compounds that showed the highest antiproliferative activity inhibited the growth of leukemia L-1210 and colon cancer SN-948 cell lines and actively controlled the proliferation of glioblastoma SNB-19, melanoma C-32, lung cancer A549, colorectal cancer Caco-2, breast cancer MCF-7 and MDA-MB231, and ductal carcinoma T47D cell lines [32,33]. Furthermore, the results of some additional experiments, such as the analysis of gene expression, indicated the induction of mitochondrial apoptosis in cancer cell lines [30,32,35,36,37].

Based on the assumption that AD and cancer have common mechanisms of formation, but differ in the reverse of processes, we decided to evaluate the cholinesterase-inhibiting activity of a set of compounds with confirmed antitumor potency. Therefore, in the present study, we searched for cholinesterase inhibitors among polycyclic, tricyclic, and tetracyclic compounds using in silico and in vitro screening methods.

## 2. Results and Discussion

In the first stage of the in silico screening process, we selected a previously synthesized library of 120 ligands, which were the derivatives of azaphenothiazine. The structures of these ligands are listed in Supplement Table S1. Most of the compounds analyzed can be assigned to one of the following two groups: diazaphenothiazines and quinobenzothiazines. Virtual screening of the compounds was performed based on the custom docking protocol that was described and validated in a previous work [38]. We tested each compound against two molecular targets: AChE in complex with *bis*-7-tacrine (PDB: 2CKM) and BuChE in complex with hydrolysis products (PDB: 1P0I). Tacrine and *bis*-7-tacrine were chosen as reference ligands due to their structural similarity with the screened compounds. Based on the results of virtual screening, we selected potential hits from each isomeric group of dipyridothiazines (1,6-diaza, 1,8-diaza, 2,7-diaza, and 3,6-diaza isomers) and 9-substituted quinobenzothiazine analogs (fluoro and methylthio derivatives). In both tricyclic and tetracyclic compounds, different substituents were attached to the nitrogen atom of the thiazine system. The values of the scoring functions achieved by the reference compounds were equal to 57.12 (tacrine) and 111.20 (*bis*-7-tacrine) after docking to 2CKM complex. During docking to BuChE, score values of 98.26 and 111.36, respectively, were reached by the compounds. The values of the evaluation function calculated for the selected compounds were in the range of 64.32–95.78 for AChE complex and 98.48–110.02 for BuChE complex.

The procedure resulted in the selection of 25 potential cholinesterase inhibitors. Compounds selected through virtual screening were tested for their ability to inhibit the activity of selected biological targets. The cholinesterase-inhibitory potency of the compounds was evaluated according to Ellman’s method [39] using AChE obtained from *E. electricus* (*ee*AChE) and BuChE obtained from *equine serum* (*eq*BuChE). Tacrine was used as a reference compound for this analysis. The results are presented in Table 1. Selected data on anticancer activity for 20 tested compounds and related references are presented in the Supplementary materials—Table S1.

All the tested compounds displayed inhibitory activity against both cholinesterases at a concentration of 10 μM during screening; however, their activity was relatively higher against *eq*BuChE. The IC_50_ values were determined for those compounds that had an inhibitory potency of greater than 50% (seven compounds in the case of *ee*AChE and 17 compounds in the case of *eq*BuChE). The results obtained revealed that among the tested compounds, there were seven nonselective inhibitors of both enzymes, two selective inhibitors of *ee*AChE, and nine selective inhibitors of *eq*BuChE. Their potencies against *ee*AChE were in a submicromolar-to-micromolar range with IC_50_ values of 0.78–11.33 μM, while their IC_50_ values against *eq*BuChE ranged from 0.46 to 10.38 μM. Such an inhibitory activity is characteristic of moderate or weak cholinesterase inhibitors. The most potent among the tested compounds were the tetracyclic ones, *N*-substituted derivatives of quinobenzothiazine (**24** and **25**) with closely related structures (*N*,*N*-diethylaminobutynyl and *N*-pyrrolidinobutynyl substituents, respectively), which inhibited both enzymes. On the other hand, *N*-substituted 9-fluoroquinobenzothiazine derivatives were found to act as selective inhibitors of *eq*BuChE, and among them, the most potent was compound **23** which showed an IC_50_ value of 0.46 μM. Since the subseries of tricyclic derivatives contain different substituents, the classical analysis of structure–activity relationships could not be performed; however, several relationships were observed to some extent. Among the tricyclic compounds tested, 1,6-diaza-isomers were found to be selective inhibitors of *eq*BuChE, except for one nonselective inhibitor (compound **2**). On the other hand, among 1,8-diaza-isomers, only one compound (compound **6**) was identified as a selective inhibitor of *eq*BuChE (IC_50_ = 0.87 μM), while the remaining compounds were nonselective inhibitors. The amino substituent was observed to have an influence on the potency of 2,7-diaza-isomers. For instance, the exchange of diethyl group with cyclic pyrrolidine substituent allowed the inhibitory potency of compound **13** to remain in the same range, while the introduction of piperidine ring improved its potency. Derivatives of 3,6-diaza-isomers were found to be rather weak inhibitors, with an exception of compound **18**. In summary, among the tested compounds, the most potent selective inhibitor of *eq*BuChE was compound **23**, and the most potent nonselective inhibitor of both enzymes was compound **24**.

**Figure 2:**
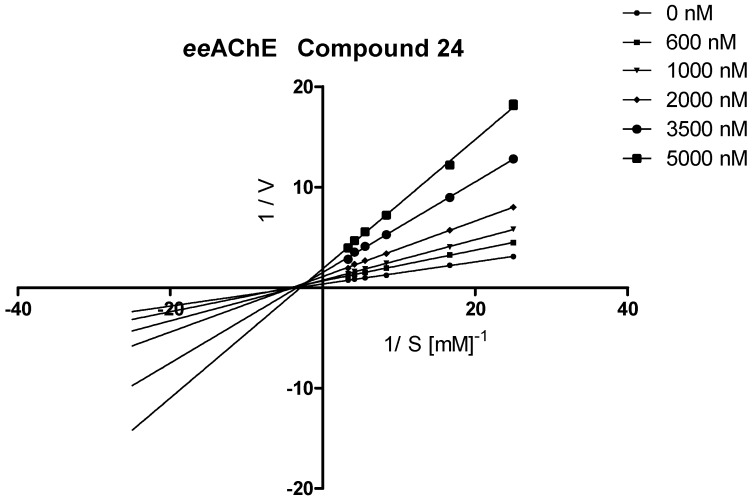
Lineweaver–Burk plot illustrating a noncompetitive type of *ee*AChE inhibition by compound **24**. S = acetylthiocholine; V = initial velocity rate.

**Figure 3:**
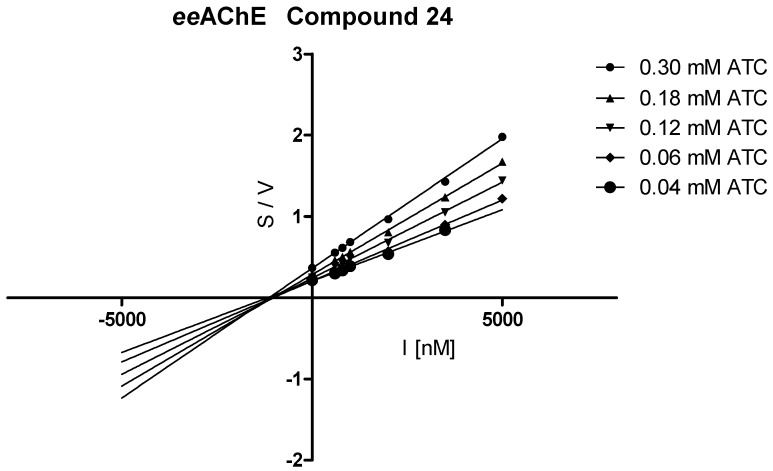
Cornish-Bowden plot illustrating a noncompetitive type of *ee*AChE inhibition by compound **24**. S = acetylthiocholine; V = initial velocity rate; I = inhibitor concentration.

**Figure 4:**
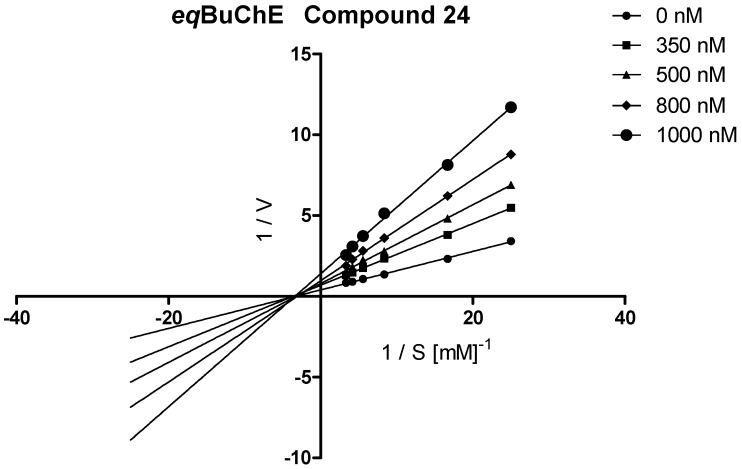
Lineweaver–Burk plot illustrating a noncompetitive type of *eq*BuChE inhibition by compound **24**. S = butyrylthiocholine; V = initial velocity rate.

**Figure 5:**
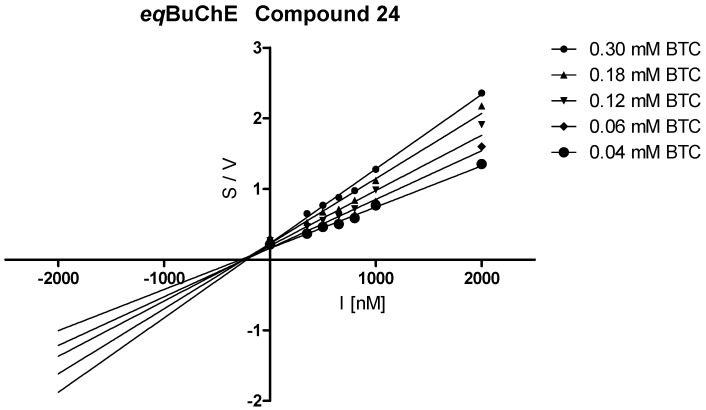
Cornish-Bowden plot illustrating a noncompetitive type of *eq*BuChE inhibition by compound **24**. S = butyrylthiocholine; V = initial velocity rate; I = inhibitor concentration.

The next step of the study was to determine the type of inhibition exhibited by the selected compound **24** which showed a good inhibitory activity against both cholinesterases. This was carried out by making use of the calculated values of Vmax and Km of the Michaelis–Menten kinetics and further through analyzing the Lineweaver–Burk and Cornish-Bowden reciprocal plots. Figure 2, Figure 3, Figure 4 and Figure 5 present the graphs that illustrate the type of inhibition exhibited by compound **24**. It can be understood that the compound displayed a noncompetitive type of inhibition against both enzymes.

**Figure 6:**
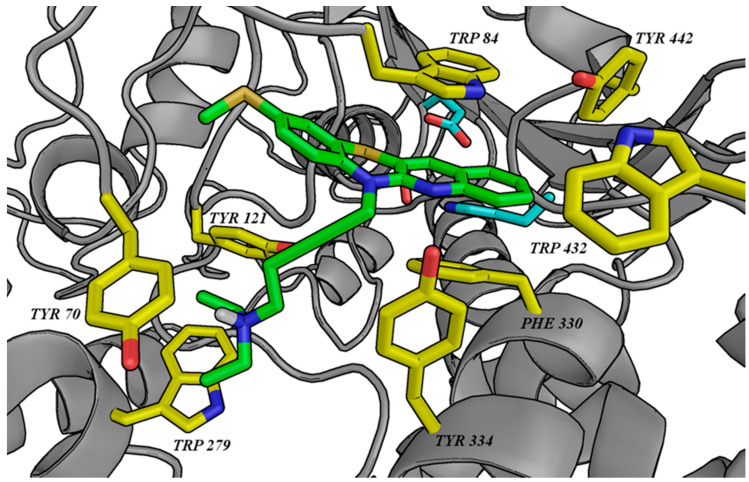
Binding mode of compound **24** in the active site of acetylcholinesterase.

The results obtained from in silico studies allow explaining the differences observed in the activity of the compounds to a certain extent. The most active inhibitor of AChE (compound **24**, Figure 6) also turned out to be the ligand that was rated the highest during screening against that enzyme.

Similar to tacrine, the tetracyclic system forms “sandwich-like” π–π interactions with TRP84 and PHE330, which were additionally reinforced by surrounding aromatic residues such as TYR334, TYR442, and TRP432. We observed similar interactions in docking results for all the tetracyclic compounds, but the example of compound **19** showed that these interactions are not enough for effective inhibition of the enzyme. The large and stiff aminobutynyl substituents directed to peripheral anionic site (PAS) were crucial for enhancing the inhibitory potential. In the cases of both quinobenzothiazine derivatives with *N*-substituted aminobutynyl moieties (**24** and **25**), tertiary amine was placed near TYR70, TYR121, and TRP279 where it can participate in the cation–π interactions. In all diazaphenothiazines with similar substituents, we observed the formation of cation–π interactions with TRP84 and PHE330. The diazaphenothiazine group was mostly directed toward the space above the oxyanion hole where we did not find its participation in either a strong or a specific interaction.

**Figure 7:**
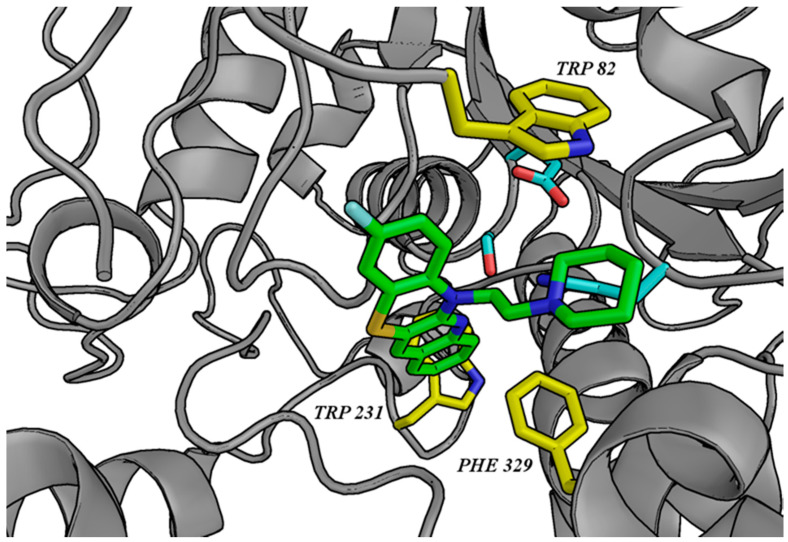
Binding mode of compound **23** in the active site of butyrylcholinesterase.

As the docking results indicated a more extensive active site for BuChE, we observed a much larger variation in the arrangement of ligands. Again, the most potent inhibitors were present among the top-scored compounds. In the case of most potent inhibitors among quinobenzothiazine derivatives, three aromatic residues (TRP82, TRP231, and PHE329) seemed to play a key role in the binding process. In this group of ligands, we most often observed CH–π interactions with TRP231 and cation–π interactions with TRP82 or PHE329. The distance between the tetracyclic moiety and the protonated nitrogen was crucial for inhibition potency of the compounds. The optimal distance in compound **23** (presented in Figure 7) allowed the abovementioned cation–π interaction to occur with both TRP82 and PHE329. In the case of diazaphenothiazine derivatives, we observed conformations interacting with the same aromatic amino acids as in the quinobenzothiazine derivatives. The differences observed in activity between particular isomers can be linked to the influence of the position of the nitrogen atom in the pyridine system on the strength of CH–π interactions.

## 3. Methods

### 3.1. Chemistry

The standard NMR spectra were recorded on a Bruker Avance spectrometer (^1^H at 600 MHz, ^13^C at 150 MHz, Bruker, Billerica, MA, USA) in CDCl_3_. Fast atom bombardment mass spectra (FAB MS, in glycerol) were performed on a Finnigan MAT 95 spectrometer (Thermo Finnigan LLC, San Jose, CA, USA) at 70 eV.

**Scheme 1:**
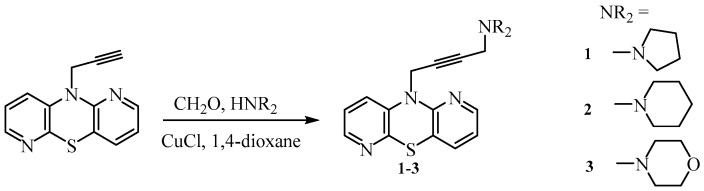
Synthesis of dipyridothiazines **1**–**3**.

Most compounds tested in the study were synthesized according to the previously described procedures: **4** and **5** [33]; **6** and **8**–**10** [34]; **7** and **11**–**13** [35]; **14**–**16** [32]; **17** and **18** [36]; **19** [40]; **20**–**23** [31]; and **24** and **25** [30]. Compounds **1**–**3** were synthesized according to Scheme 1 as follows.

A mixture of 10-propargyl-1,6-diazaphenothiazine (120.5 mg, 0.5 mmol), paraformaldehyde (50 mg, 0.5 mmol), amine (0.7 mmol), and cuprous chloride (catalytic amount) in peroxide-free, dry 1,4-dioxane (10 mL) was heated with continuous stirring at 70 °C for 3 h. After cooling (20 mL) water was added and the mixture was extracted with chloroform (50 mL), dried with Na_2_SO_4_, and evaporated in vacuo. The dry residue was dissolved in CHCl_3_ and purified by column chromatography (aluminium oxide, CHCl_3_) to obtain compounds **1**–**3**.

#### 3.1.1. 10-(4-Pyrrolidin-1-yl-but-2-ynyl)-1,6-diazaphenothiazine (**1**)

(0.121 g, 76%); an oil. 1H NMR (CDCl_3_) δ: 1.761–.79 (m, 4H, 2CH_2_), 2.552–.57 (m, 4H, 2CH_2_), 3.41 (s, 2H, CH_2_) 4.71 (s, 2H, CH_2_), 6.83 (dd, *J* = 7.2 Hz, *J* = 4.8 Hz, 1H, H3), 7.04 (d, *J* = 7.8 Hz, 1H, H9), 7.307–.34 (m, 2H H8, H4), 8.018–.05 (m, 2H, H2, H7). ^13^C NMR (CDCl_3_) δ: 23.77, 35.17, 43.32, 52.60, 79.18, 80.32, 116.28, 118.51,121.24, 121.87, 134.56, 137.90, 142.96, 144.51, 145.22, 151.97. FAB MS *m/z*: 322 (M, 80), 252 (M+1-C_4_H_8_N, 100). TLC Anal.: (aluminum oxide 60F_254_ neutral, CHCl_3_) R_f_ = 0.33. Anal. Calcd for: C_18_H_18_N_4_S C, 67.05, H 5.63, N 17.38. Found: C, 66.84, H 5.58, N 17.11.

#### 3.1.2. 10-(4-Piperidin-1-yl-but-2-ynyl)- 1,6-diazaphenothiazine (**2**)

(0.124 g, 74%); an oil. 1H NMR (CDCl_3_) δ: 1.401–.42 (m, 2H, CH_2_), 1.591–.61 (m, 4H, 2CH_2_), 2.462–.48 (m, 4H, 2CH_2_), 3.28 (s, 2H, CH_2_), 4.72 (s, 2H, CH_2_), 6.79 (dd, *J* = 7.2 Hz, *J* = 4.8 Hz, 1H, H3), 7.05 (d, *J* = 7.8 Hz, 1H, H9), 7.317–.34 (m, 2H H8, H4), 8.028–.04 (m, 2H, H2, H7). ^13^C NMR (CDCl_3_) δ: 23.83, 25.75, 35.17, 53.27, 79.80, 80.10, 116.28, 118.51,121.33, 121.86, 134.57, 134.63, 137.90, 142.95, 144.50, 145.22, 151.98. FAB MS *m/z*: 337 (M, 70), 201 (M+1-C_5_H_10_N, 100). TLC Anal.: (aluminum oxide 60F_254_ neutral, CHCl_3_) R_f_ = 0.41. Anal. Calcd for: C_19_H_20_N_4_S C, 67.83, H 5.99, N 16.65. Found: C, 67.59, H 5.91, N 16.40.

#### 3.1.3. 10-(4-Morpholin-4-yl-but-2-ynyl)-1,6-diazaphenothiazine (**3**)

(0.115 g, 68%); an oil. 1H NMR (CDCl_3_) δ: 1.71–.78 (m, 2H, CH_2_), 2.552–.58 (m, 2H, CH_2_), 3.403–.43 (m, 4H, 2CH_2_), 4.71 (s, 2H, CH_2_), 6.78 (dd, *J* = 7.2 Hz, *J* = 4.8 Hz, 1H, H3), 7.05 (d, *J* = 7.8 Hz, 1H, H9), 7.287–.31 (m, 2H, H8, H4), 8.018–.05 (m, 2H, H2, H7). ^13^C NMR (CDCl_3_) δ: 23.81, 35.20, 43.41, 52.63, 79.18, 80.37, 116.28, 118.51,121.24, 121.87, 134.56, 137.90, 142.96, 144.51, 145.22, 151.97.FAB MS *m/z*: 339 (M+1, 40), 201 (M+1-C_8_H_12_NO, 60). TLC Anal.: (aluminum oxide 60F_254_ neutral, CHCl_3_) R_f_ = 0.38. Anal. Calcd for: C_18_H_18_N_4_SO C 63.88, H 5.36, N 16.56. Found: C 63.68, H 5.31, N 16.44.

### 3.2. Molecular Modeling

The three-dimensional structure of potential cholinesterase inhibitors (120 ligands) was drawn in Corina on-line (Molecular Networks and Altamira) [41]. Atom types were checked, hydrogen atoms were added, and Gasteiger–Marsili charges were assigned with Sybyl X 2.1 (Tripos, Certara, Princeton, NJ, USA) [42]. AChE and BuChE were prepared from the crystal structures of 2CKM and 1P0I, respectively, before virtual screening in Hermes (CCDC). Histidine residues were protonated at Nε, and hydrogen atoms were added. Water molecules and ligands were removed from the 2CKM complex, whereas in 1P0I, 24 water molecules were saved using toggle option thereby allowing the program to decide to use them in calculations. Docking was performed with GoldSuite 5.1 (CCDC) [43]. We defined binding sites as all amino acid residues present within a radius of 10 Å from *bis*-(7)-tacrine in the case of AChE and 20 Å from the glycerol molecule in the case of BuChE. The genetic algorithm was started with automatic settings for flexible ligands. As a result, we obtained three top-scored ligand poses per compound, which were sorted by GoldScore and ChemScore function values for AChE and BuChE, respectively. The results were visualized using PyMol 0.99rc6 [44]. The whole procedure was already described and validated [38].

### 3.3. Biological Tests

#### 3.3.1. AChE/BuChE-Inhibitory Activity

The following reagents were purchased from Sigma–Aldrich (Steinheim, Germany): 5,5′-dithiobis-(2-nitrobenzoic acid) (DNTB), acetylthiocholine iodide (ATC), butyrylthiocholine iodide (BTC), AChE from *Electrophorus electricus*, and BuChE from horse serum. The enzymes were prepared as aqueous stock solutions with a concentration of 5 U/mL. Before use, they were diluted to a final concentration of 3.125 U/mL. In addition, solutions of 0.0125 M DTNB, 0.01875 M ATC, and 0.01875 M BTC, as well as 0.1 M phosphate buffer (pH 8.0), were prepared in water. The biological assay was performed using Ellman’s method [39] which was modified for 24-well plates. For this assay, 25 µL of the tested compounds or water (in the blank samples) and 20 µL of the enzyme in 765 µL of the buffer were incubated for 5 min at 25 °C before starting the reaction. Then, 20 µL of DTNB and 20 µL of the ATC/BTC solutions were added. After 5 min, the changes in absorbance were measured at 412 nm using an EnSpire multimode microplate reader (PerkinElmer, Waltham, MA, USA). All the compounds were tested at a screening concentration of 10 μM. For determining the enzyme inhibition, the following formula was used: 100 − (S/B) × 100, where S and B were the activities of the respective enzymes with and without the test sample, respectively. IC_50_ values were determined for compounds that showed greater than 50% inhibitory activity at the screening concentration. To determine the IC_50_ value, the absorbance measured at six different concentrations of the inhibitor was converted to % inhibition of the enzyme and plotted against the applied concentration of the inhibitor, using nonlinear regression (GraphPad Prism 5; GraphPad Software, San Diego, CA, USA). Tacrine was used as a reference compound for the assay. All the reactions were performed in triplicate. Data are expressed as the mean ± SEM.

#### 3.3.2. Kinetic Studies

Kinetic studies were carried out by following Ellman’s method [39] modified for 96-well plates, using different concentrations of the substrate. The stock solutions of ATC and BTC (0.02125 M) were prepared in water and diluted before use. The aqueous stock solutions of enzymes (5 U/mL) were diluted to a final concentration of 0.384 U/mL. For each concentration of the test compounds, ATC or BTC was used at a concentration of 0.3, 0.24, 0.18, 0.12, 0.06, and 0.04 mM in the wells. Lineweaver–Burk and Cornish-Bowden plots were generated using linear regression in GraphPad Prism 5 (GraphPad Software, San Diego, CA, USA). Each experiment was performed in triplicate.

## 4. Conclusions

The in silico and in vitro tests performed in this study led to the identification of cholinesterase inhibitors among dipyridothiazine and quinobenzothiazine derivatives. Compounds with dual antitumor and cholinesterase-inhibitory properties can be useful both in the treatment of cancer and AD prevention [45]. Such a conclusion can be drawn based on the literature reports indicating a lower incidence of AD among people who have previously received chemotherapy. In the case of AD, neuropathological changes, including loss of cholinergic neurons and the formation of Aβ plaques and NFTs, which lead to the development of disease symptoms, probably appear several years earlier. Hence, disease treatment may begin too late and it may be difficult to find an effective AD therapy because no prophylactic drugs have been used before the onset of symptoms. It can, therefore, be assumed that some anticancer drugs may exert a preventive effect by inhibiting the neurodegenerative processes at an earlier stage of disease development. Dual antitumor and cholinesterase-inhibitory activity can be considered as a valuable combination for the treatment of both cancer and AD. Such dual properties are found in the compounds described herein, and the results of this study might open new directions of research on the group of tricyclic phenothiazine derivatives.

## Figures and Tables

**Table 1:** Inhibition of AChE and BuChE by polycyclic compounds selected by virtual in silico screening.

Compound		R	% Inhibition +/− Ϭ ^a^	IC_50_ *ee*AChE ^b^ [µM] +/− SEM ^c^	% Inhibition +/− Ϭ	IC_50_ *eq*BuChE ^d^ [µM] +/− SEM
**Dipyridothiazines**						
isomer 1,6-diaza 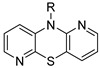	**1**	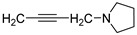	45.4 ± 9.8	—	65.4 ± 3.0	5.748 ± 0.164
	**2**	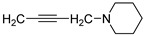	60.3 ± 8.8	8.309 ± 0.315	56.6 ± 2.4	8.942 ± 0.239
	**3**	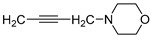	13.7 ± 1.5	—	63.6 ± 2.4	9.250 ± 0.275
	**4**	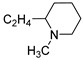	22.4 ± 1.1	—	57.0 ± 7.4	9.493 ± 0.330
	**5**	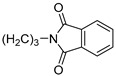	7.3 ± 0.9	—	52.5 ± 5.9	10.080 ± 0.441
isomer 1,8-diaza 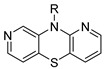	**6**	(CH_2_)_3_N(CH_3_)_2_	20.4 ± 3.6	—	85.8 ± 0.5	0.865 ± 0.011
	**7**	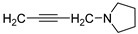	57.3 ± 0.4	10.020 ± 0.604	91.1 ± 0.8	0.929 ± 0.016
	**8**	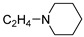	33.5 ± 1.6	—	66.0 ± 3.4	4.442 ± 0.049
	**9**	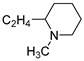	58.1 ± 4.1	6.546 ± 0.177	76.4 ± 0.2	3.034 ± 0.072
	**10**	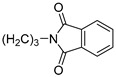	49.6 ± 1.3	9.410 ± 0.489	71.9 ± 4.3	3.666 ± 0.054
isomer 2,7-diaza 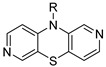	**11**	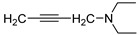	20.1 ± 1.4	—	20.7 ± 0.9	—
	**12**	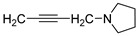	20.3 ± 1.0	—	31.4 ± 1.7	—
	**13**	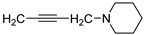	51.6 ± 3.2	11.330 ± 0.464	37.6 ± 1.6	—
isomer 3,6-diaza 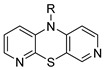	**14**	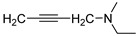	7.7 ± 2.1	—	20.4 ± 1.9	—
	**15**	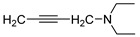	10.1 ± 3.1	—	17.2 ± 7.3	—
	**16**	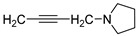	21.9 ± 5.7	—	58.5 ± 9.0	10.380 ± 0.308
	**17**	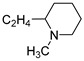	33.0 ± 4.5	—	16.1 ± 0.7	—
	**18**	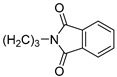	72.2 ± 0.7	4.263 ± 0.078	30.5 ± 5.7	—
***N*-substituted 9-fluoroquinobenzothiazines** 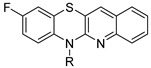						
	**19**	H	6.6 ± 1.3	—	81.0 ± 1.4	1.020 ± 0.028
	**20**	CH_3_	3.0 ± 1.6	—	35.3 ± 12.3	—
	**21**	(CH_2_)_3_NH_2_	6.5 ± 2.8	—	76.3 ± 1.2	2.511 ± 0.047
	**22**	(CH_2_)_4_NH_2_	2.3 ± 2.1	—	64.7 ± 1.0	3.929 ± 0.196
	**23**	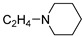	27.0 ± 2.8	—	97.7 ± 0.5	0.463 ± 0.010
***N*-substituted 9-methylthioquinobenzothiazines** 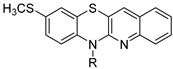						
	**24**	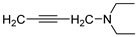	83.4 ± 1.1	0.784 ± 0.018	95.5 ± 0.8	0.535 ± 0.013
	**25**	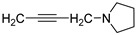	78.0 ± 1.4	2.218 ± 0.062	99.9 ± 0.7	0.506 ± 0.014
**Reference**						
Tacrine				0.024 ± 0.001		0.002 ± 0.0005

^a^ % of inhibition at 10 µM compound concentration; values are expressed as mean ± standard deviation (Ϭ); ^b^ acetylcholinesterase from *electric eel*; ^c^ IC_50_ values, expressed as mean ± standard error of the mean (SEM) of at least three experiments; ^d^ butyrylcholinesterase form *equine serum*.

## Supplementary Materials

The following are available online. Table S1. Structures of tested compounds.
