# Supplementary material for: Dual Action of Dipyridothiazine and Quinobenzothiazine Derivatives—Anticancer and Cholinesterase-Inhibiting Activity
Source: Molecules. 2020 Jun 3;25(11):2604. doi: 10.3390/molecules25112604 (PMC7321178; doi:10.3390/molecules25112604)

Supplementary Material

Dual action of dipyridothiazine and quinobenzothiazine derivatives – anticancer and cholinesterase- inhibiting activity

Jakub Jończyk[1], Justyna Godyń[1], Ewelina Stawarska[1], Beata Morak-Młodawska[2], Małgorzata Jeleń[2], Krystian Pluta[2], Barbara Malawska[1*]

[1]Department of Physicochemical Drug Analysis, Faculty of Pharmacy, Jagiellonian University Medical College, Kraków 30-688, Poland

[2]The Medical University of Silesia in Katowice, Faculty of Pharmaceutical Sciences in Sosnowiec, Department of Organic Chemistry, Jagiellońska 4, 41-200 Sosnowiec, Poland,

Content

1. **Table S1**. Structures of tested compounds.
2. **Table S2**. Effects of azaphenothiazines **1-25** on the cancer cell lines and phytohemagglutinin-induced blood lymphocyte proliferation
3. ^{1}H NMR of compound **1**
4. ^{13}C NMR of compound **1**
5. FAB MS of compound **1**
6. ^{1}H NMR of compound **2**
7. ^{13}C NMR of compound **2**
8. FAB MS of compound **2**
9. ^{1}H NMR of compound **3**
10. ^{13}C NMR of compound **3**
11. FAB MS of compound **3**

Table S1. Structures of tested compounds.

Dipyridothiazines	**Compound**	**R**
isomer 2,7-diaza		
	**S1**	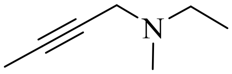
	**S2 (11)**	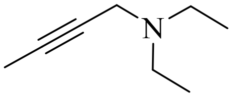
	**S3 (12)**	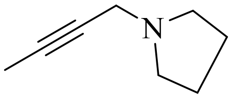
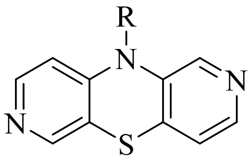	**S4 (13)**	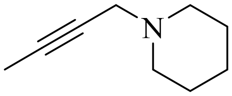
	**S5**	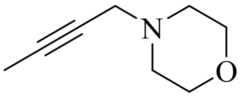
	**S6**	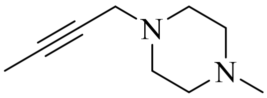
	**S7**	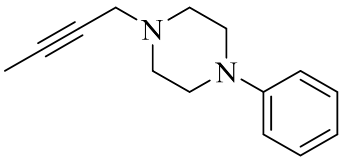
isomer 1,8-diaza		
	**S8**	**H**
	**S9**	**CH_3**
	**S10**	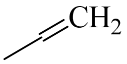
	**S11**	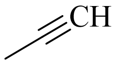
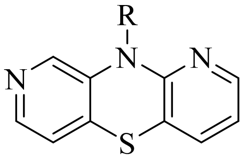	**S12**	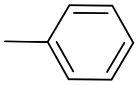
	**S13**	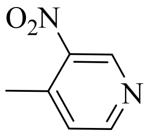
	**S14**	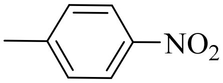
	**S15 (6)**	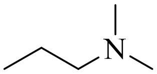
	**S16**	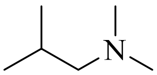

S17

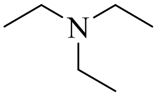

S18

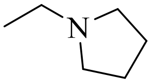

S19 (9)

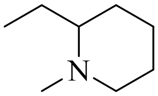

S20 (8)

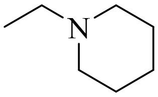

S21

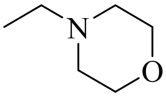

S22 (10)

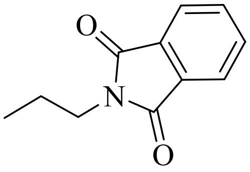

S23

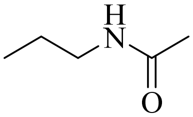

S24

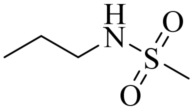

S25

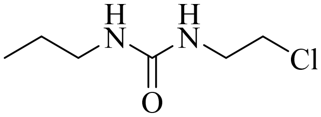

S26

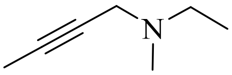

S27

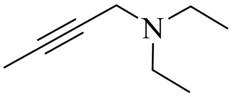

S28 (7)

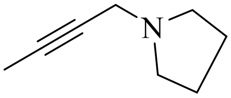

S29

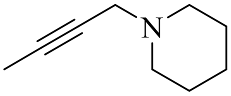

S30

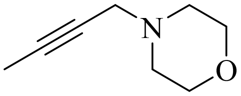

S31

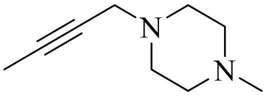

S32

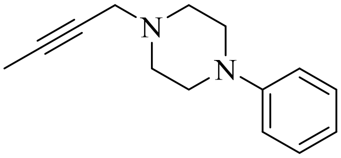

isomer 3,6-diaza

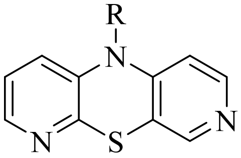

**S33**	**H**
**S34**	**CH_3**
**S35**	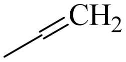
**S36**	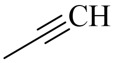
**S37**	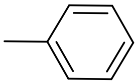
**S38**	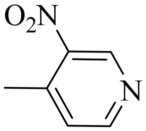
**S39**	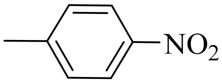
**S40**	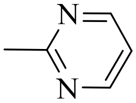
**S41**	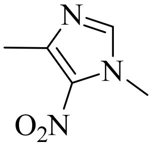
**S42**	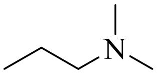
**S43**	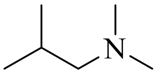
**S44**	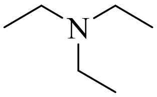
**S45**	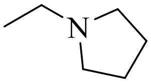
**S46 (17)**	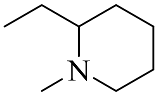
**S47**	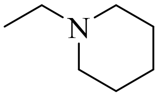
**S48**	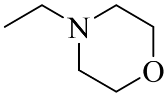
**S49 (18)**	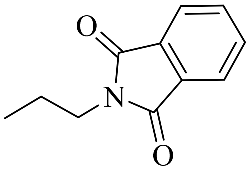

S50 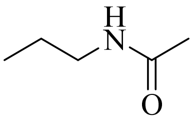

S51 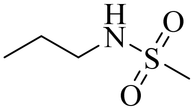

S52 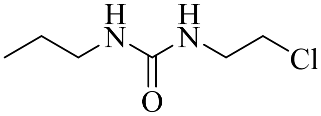

S53 (14) 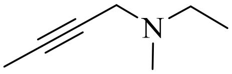

S54 (15) 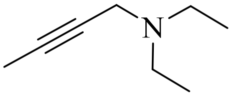

S55 (16) 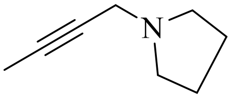

S56 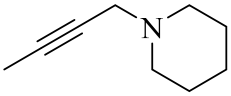

S57 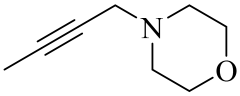

S58 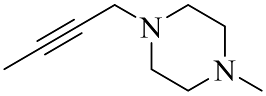

S59 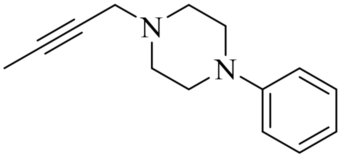

isomer 1,6-diaza

S60 **H**

S61 **CH_3**

S62 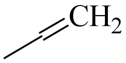

S63 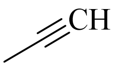

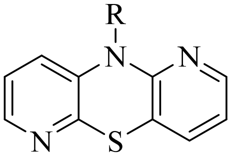

S64 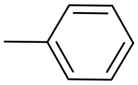

S65 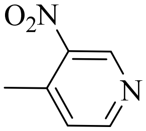

S66 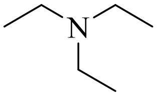

S67 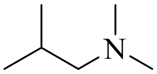

Compound	R
**S68**	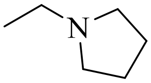
**S69**	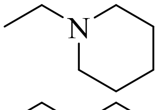
**S70 (4)**	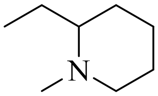
**S71**	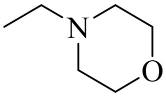
**S72 (5)**	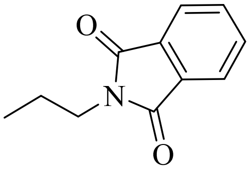
**S73**	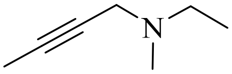
**S74**	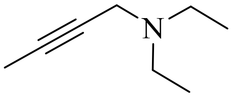
**S75 (1)**	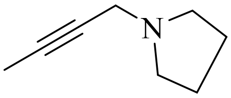
**S76 (2)**	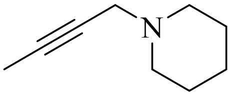
**S77 (3)**	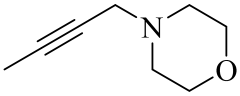
**S78**	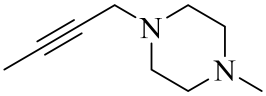
**S79**	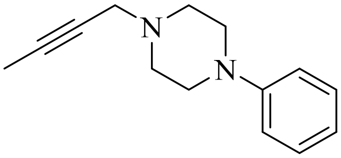

N-substituted quinobenzothiazines

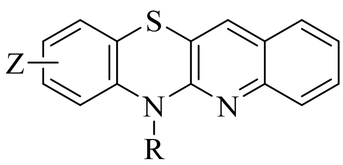

**Compound**	**Z**	**R**
**S80 (19)**	**9-F**	**H**
**S81 (20)**	**9-F**	**CH_3**
**S82**	**9-F**	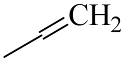
**S83**	**9-F**	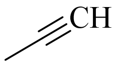
**S84**	**9-F**	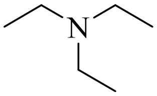
**S85**	**9-F**	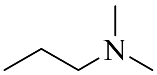

**S86**	**9-F**	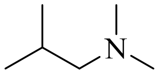
**S87**	**9-F**	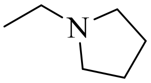
**S88 (23)**	**9-F**	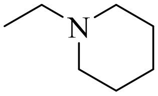
**S89**	**9-F**	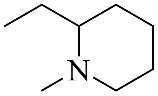
**S90**	**9-F**	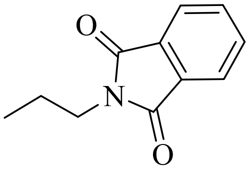
**S91 (21)**	**9-F**	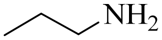
**S92**	**9-F**	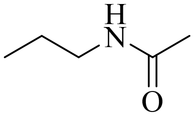
**S93**	**9-F**	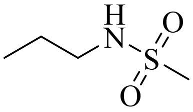
**S94**	**9-F**	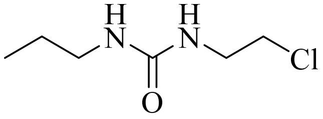
**S95**	**9-F**	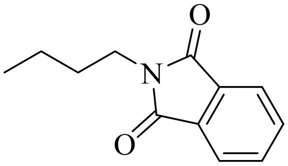
**S96 (22)**	**9-F**	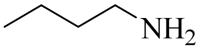
**S97**	**9-F**	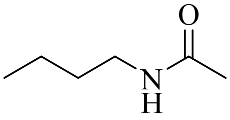
**S98**	**9-F**	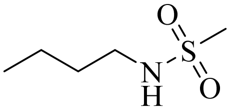
**S99**	**9-F**	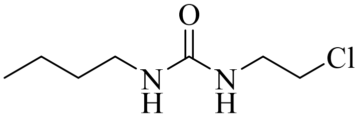
**S100**	**8-Cl**	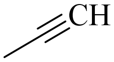
**S101**	**9-Cl**	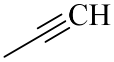
**S102**	**9-Br**	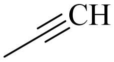

	**S103**	**9-CF_3**	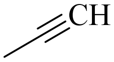
	**S104**	**9-OCH_3**	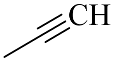
	**S105**	**9-SCH_3**	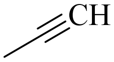
	**S106**	**10-Cl**	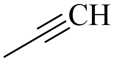
	**S107 (24)**	**9-SCH_3**	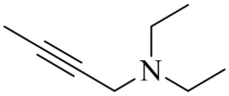
	**S108 (25)**	**9-SCH_3**	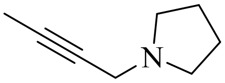
	**S109**	**9-SCH_3**	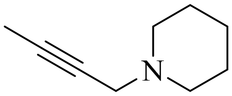

N-substituted quinaphthaphthiazines	**Compound**	**R**
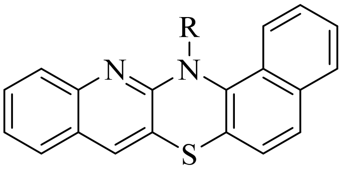	**S110**	H
	**S111**	CH_3
	**S112**	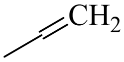
	**S113**	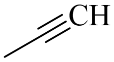
	**S114**	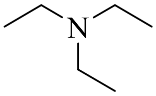

	**Compound**	**R**
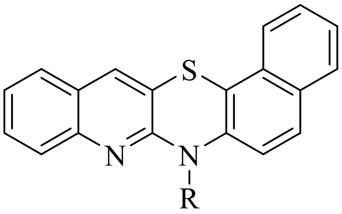	**S115**	H
	**S116**	CH_3
	**S117**	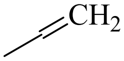
	**S118**	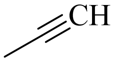
	**S119**	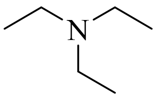

N-substituted diquinothiazine	**Compound**	**R**

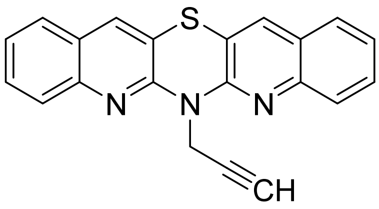

S120 –

Table S2. Effects of azaphenothiazines **1-25** on the cancer cell lines and phytohemagglutinin-induced blood lymphocyte (PBMC) proliferation

No	Anticancer and antiproliferative activity [(IC_{50} in µM and inhibition in % (µM)]	Ref.
**1**	No tested	-
**2**	No tested	-
**3**	No tested	-
**4**	SNB-19 96.0; C-32 50.0; MCF-7 28.8	33
**5**	SNB-19 > 100; C-32 > 100; MCF-7 21.1	33
**6**	L-1210 19.2; SV948 47.6	34
**7**	SNB-19 > 100; C-32 > 100; T47D >100	35
**8**	PBMC 0.5% (32.0)	34
**9**	PBMC 7.0% (30.7)	34
**10**	PBMC 12.5% (25.8)	34
**11**	SNB-19 > 100; C-32 100; T47D > 100	35
**12**	SNB-19 > 100; C-32 > 100; T47D > 100	35
**13**	SNB-19 > 100; C-32 73.8; T47D > 100	35
**14**	SNB-19 0.35; C-32 4.9; MDA-MB231 9.1	32
**15**	SNB-19 0.34; C-32 1.7; MDA-MB231 19.5	32
**16**	SNB-19 > 100; C-32 > 100; MDA-MB231 > 100	32
**17**	SNB-19 > 100; C-32 > 100; MCF-7 87.1	36
**18**	SNB-19 > 100; C-32 > 100; MCF-7 > 100	36
**19**	PBMC 100% (37.3)	37
**20**	PBMC 27.8% (3.5)	31
**21**	No tested	-
**22**	No tested	-
**23**	PBMC 88.4% (2.6)	31
**24**	PBMC 94.2% (23.9)	30
**25**	PBMC 92.2% (24.0)	30

3. ¹H NMR of compound **1**

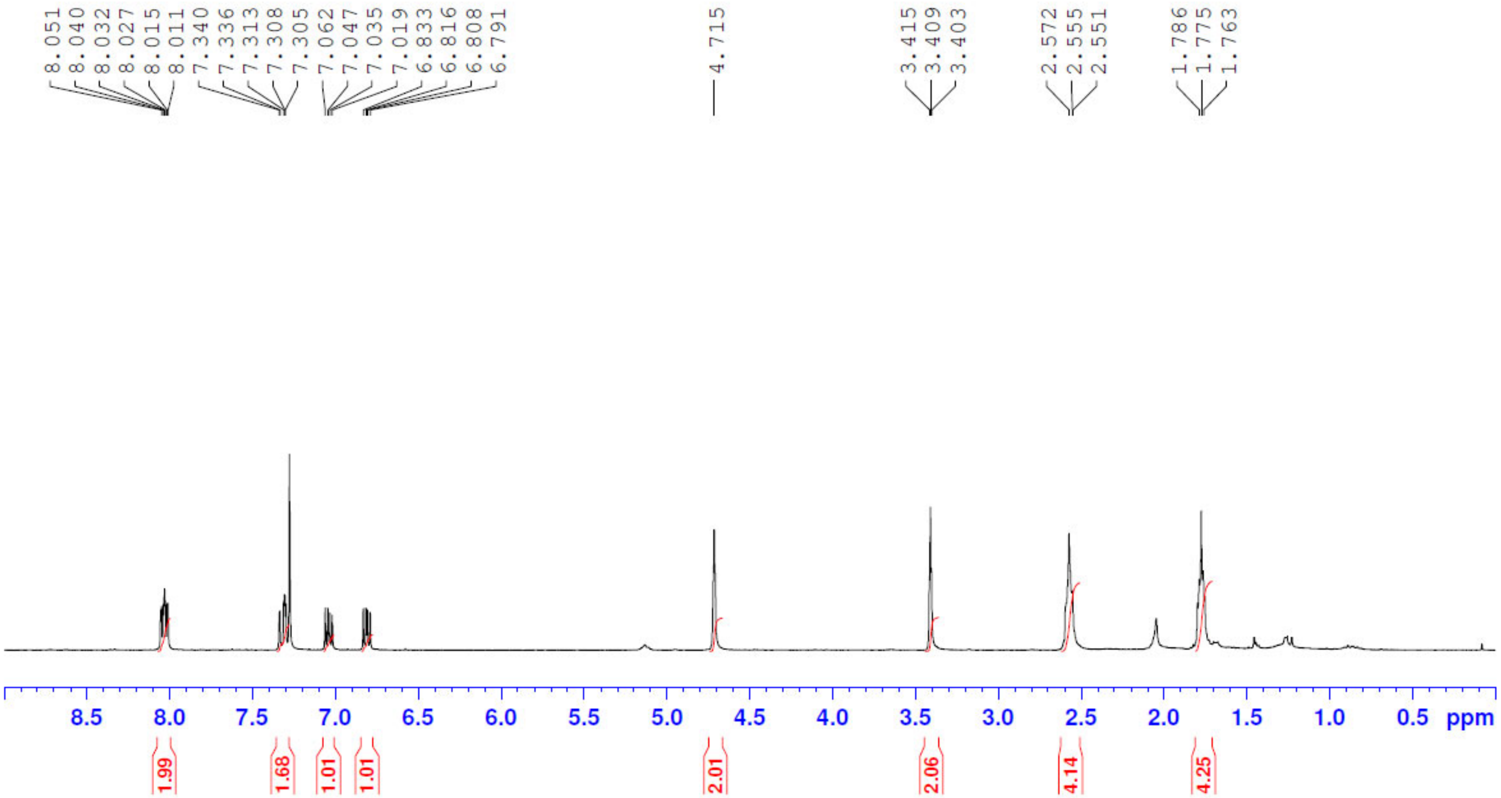

4. ^{13}C NMR of compound **1**

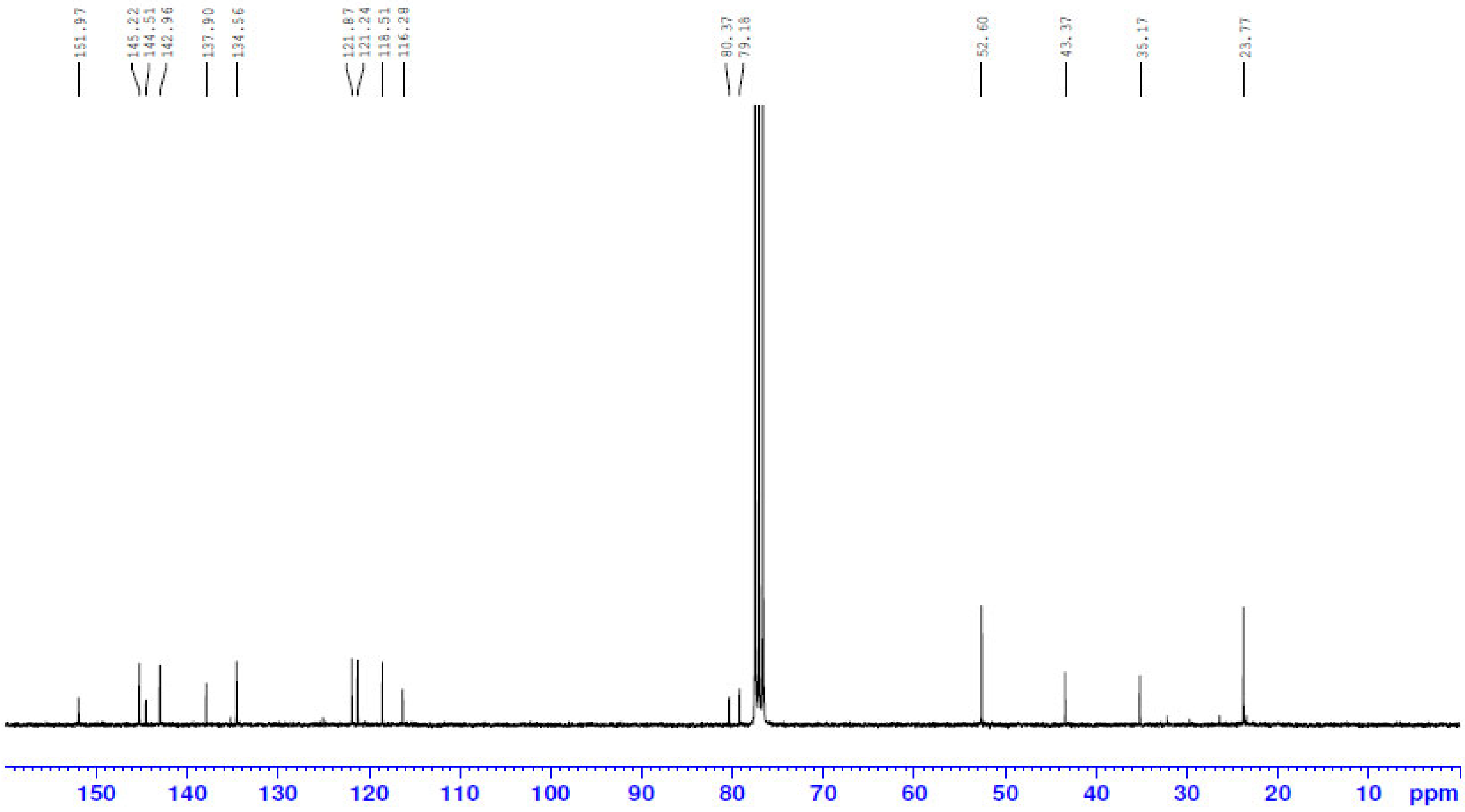

5. FAB MS of compound **1**

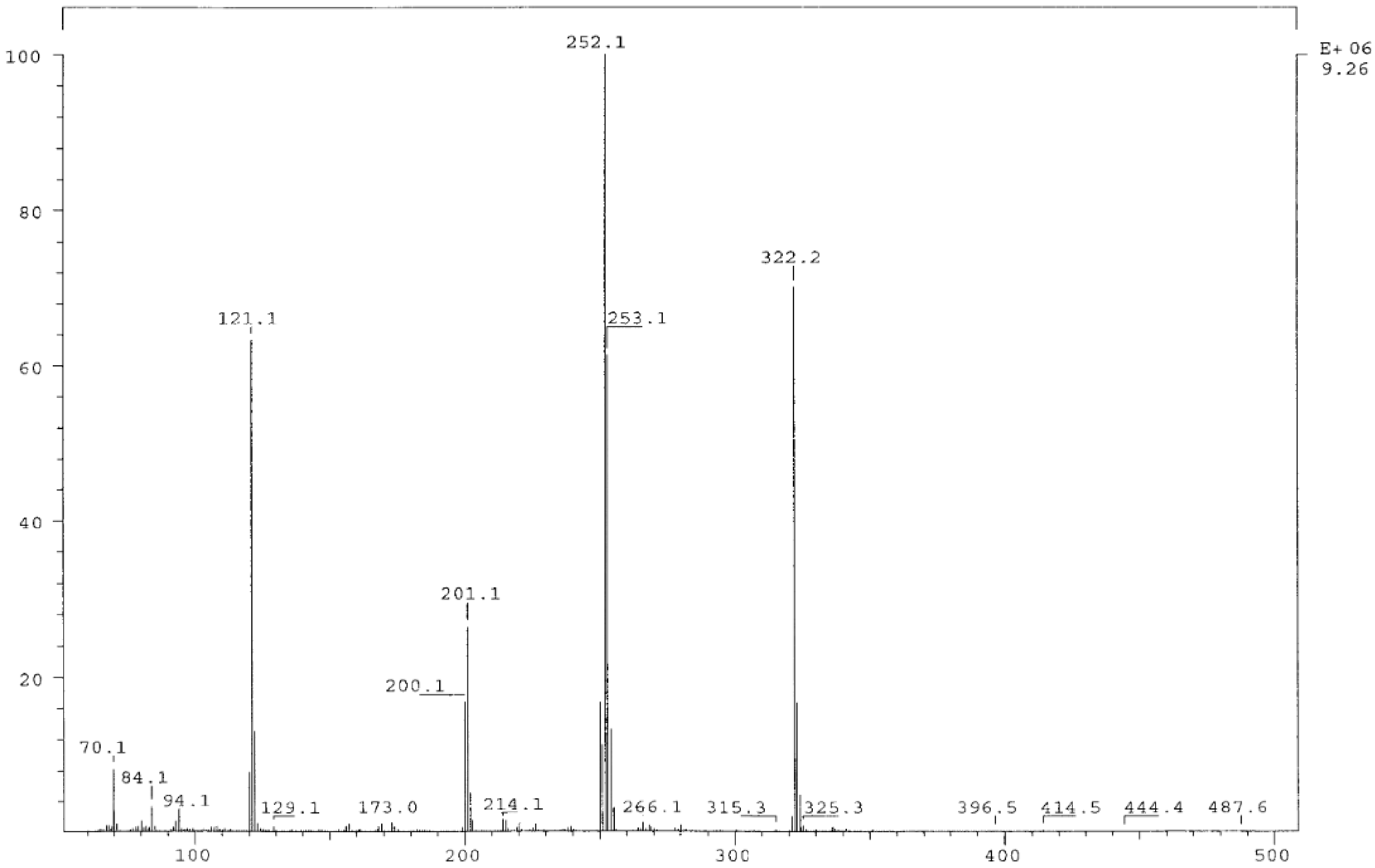

6. ¹H NMR of compound **2**

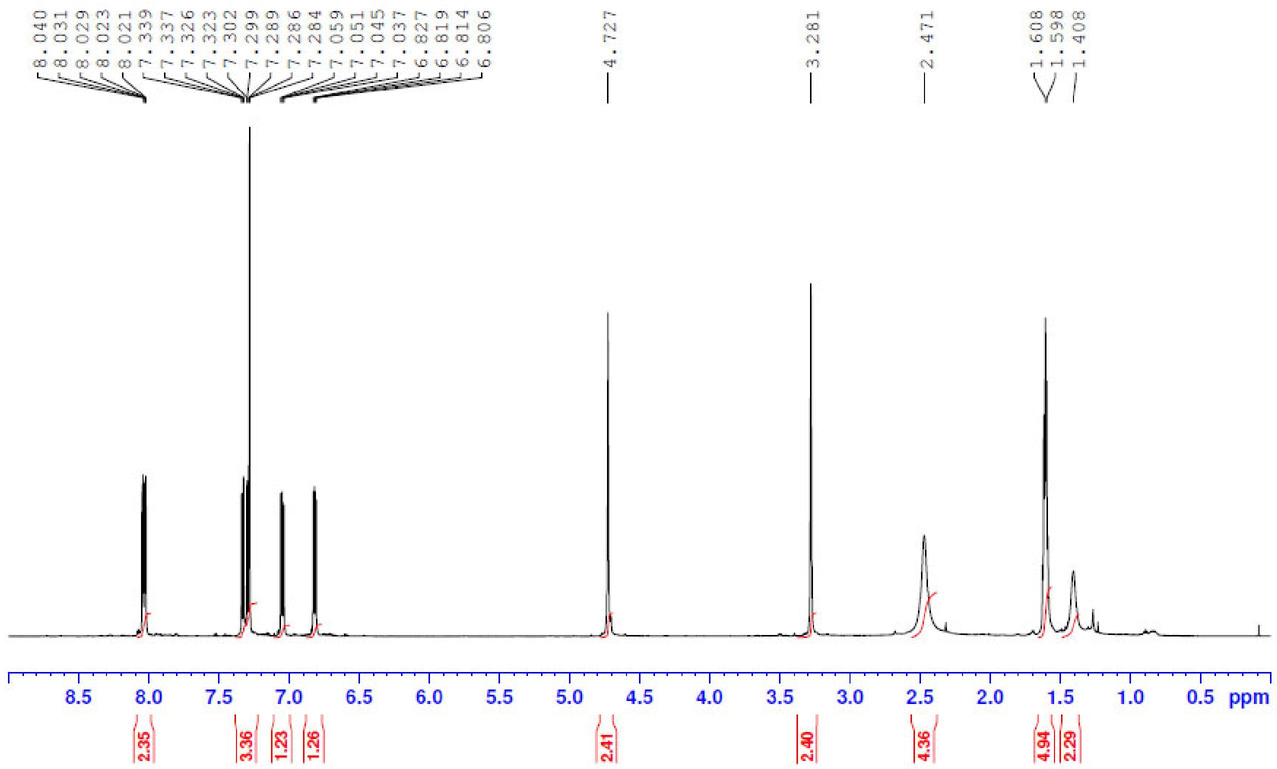

7. ¹³C NMR of compound **2**

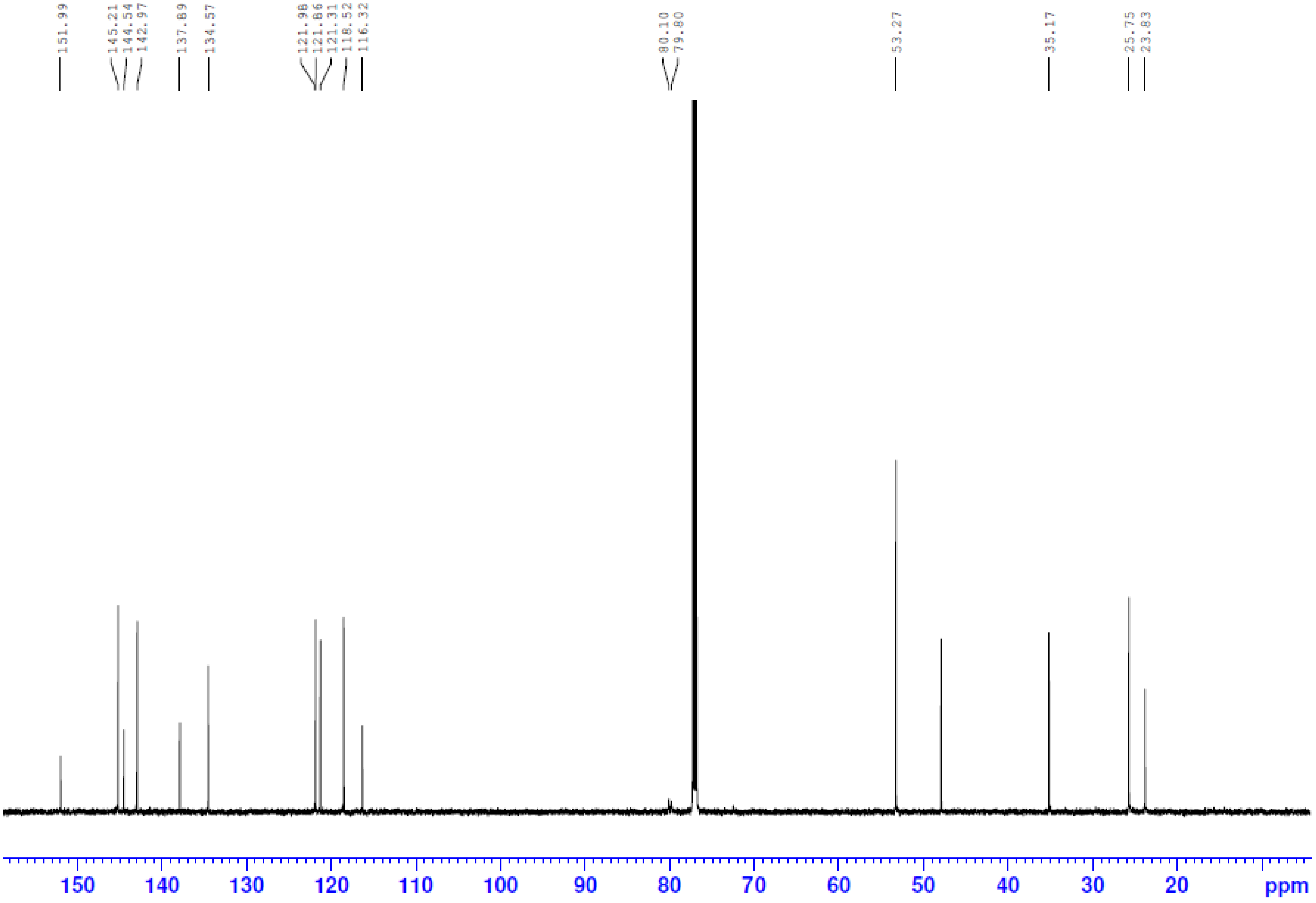

8. FAB MS of compound **2**

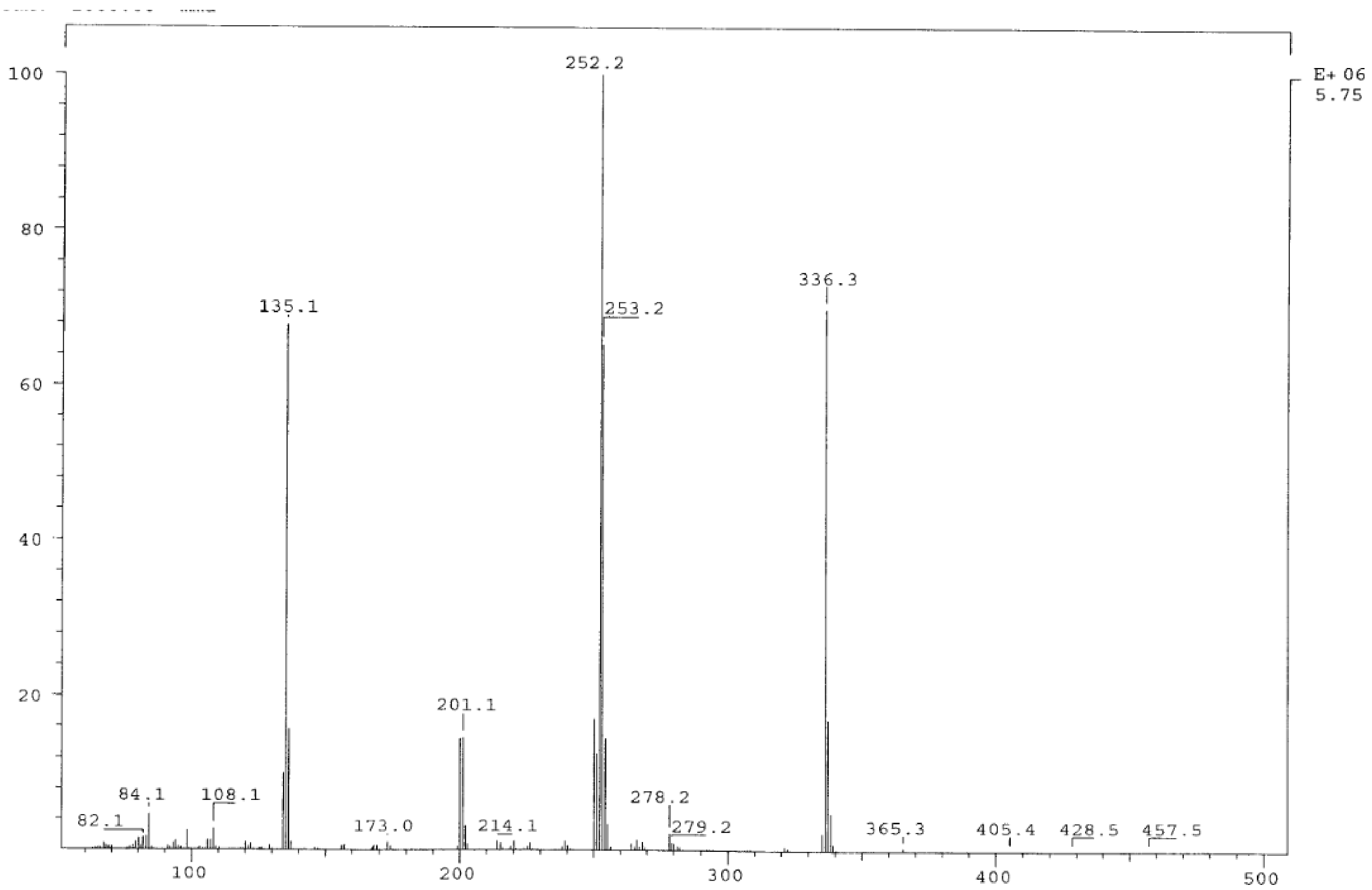

9. ^{1}H NMR of compound **3**

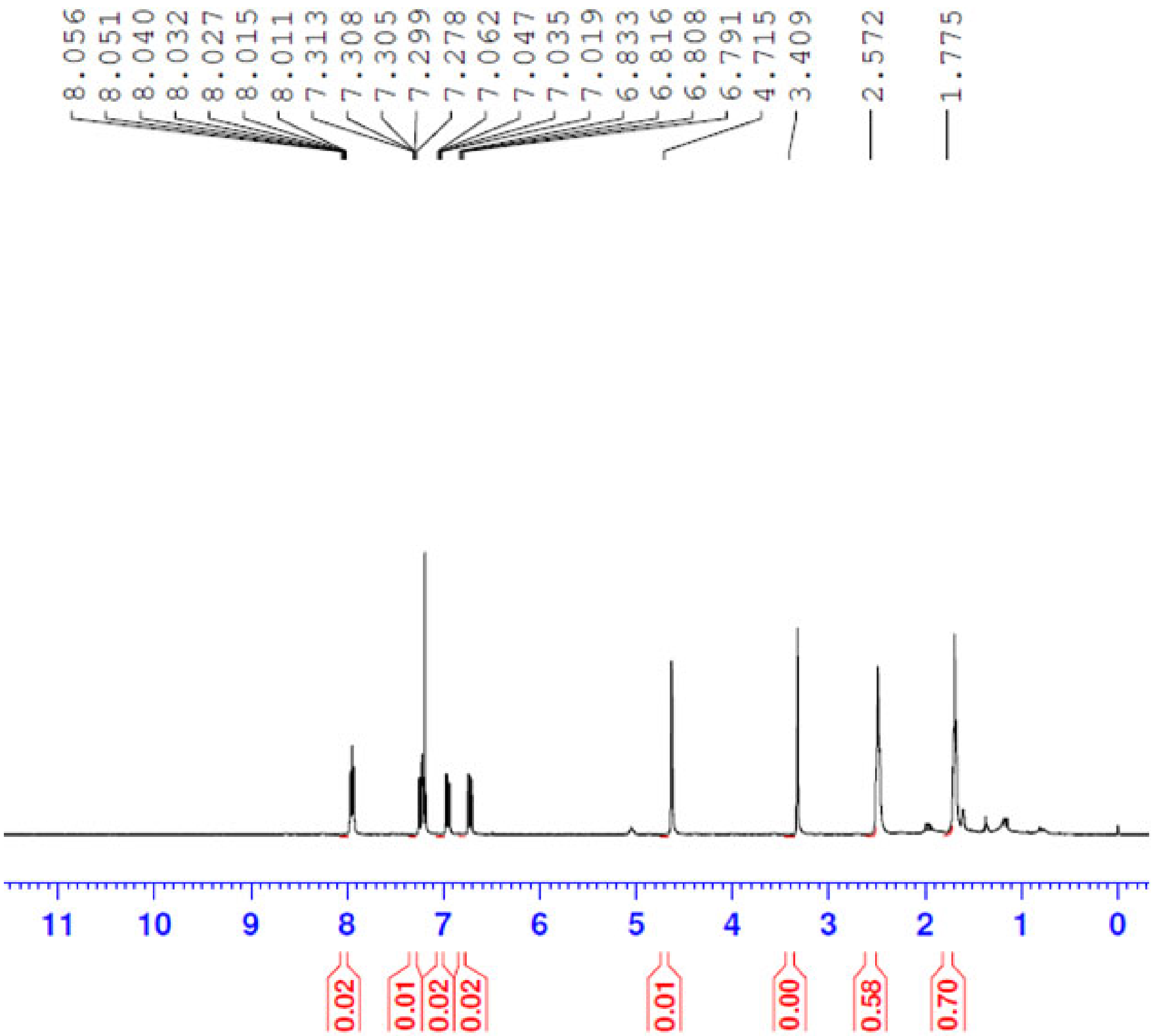

10. ¹³C NMR of compound **3**

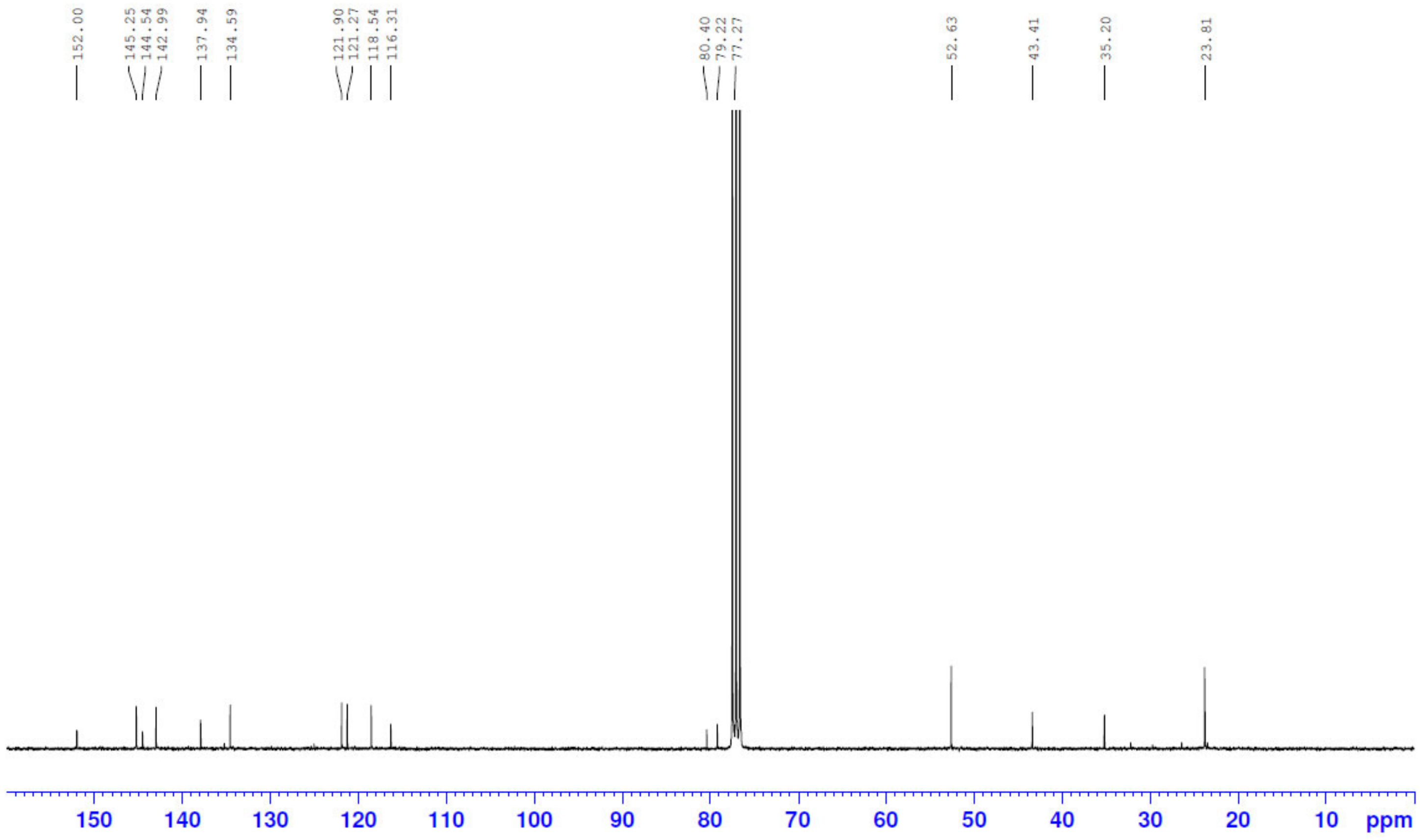

11. FAB MS of compound **3**

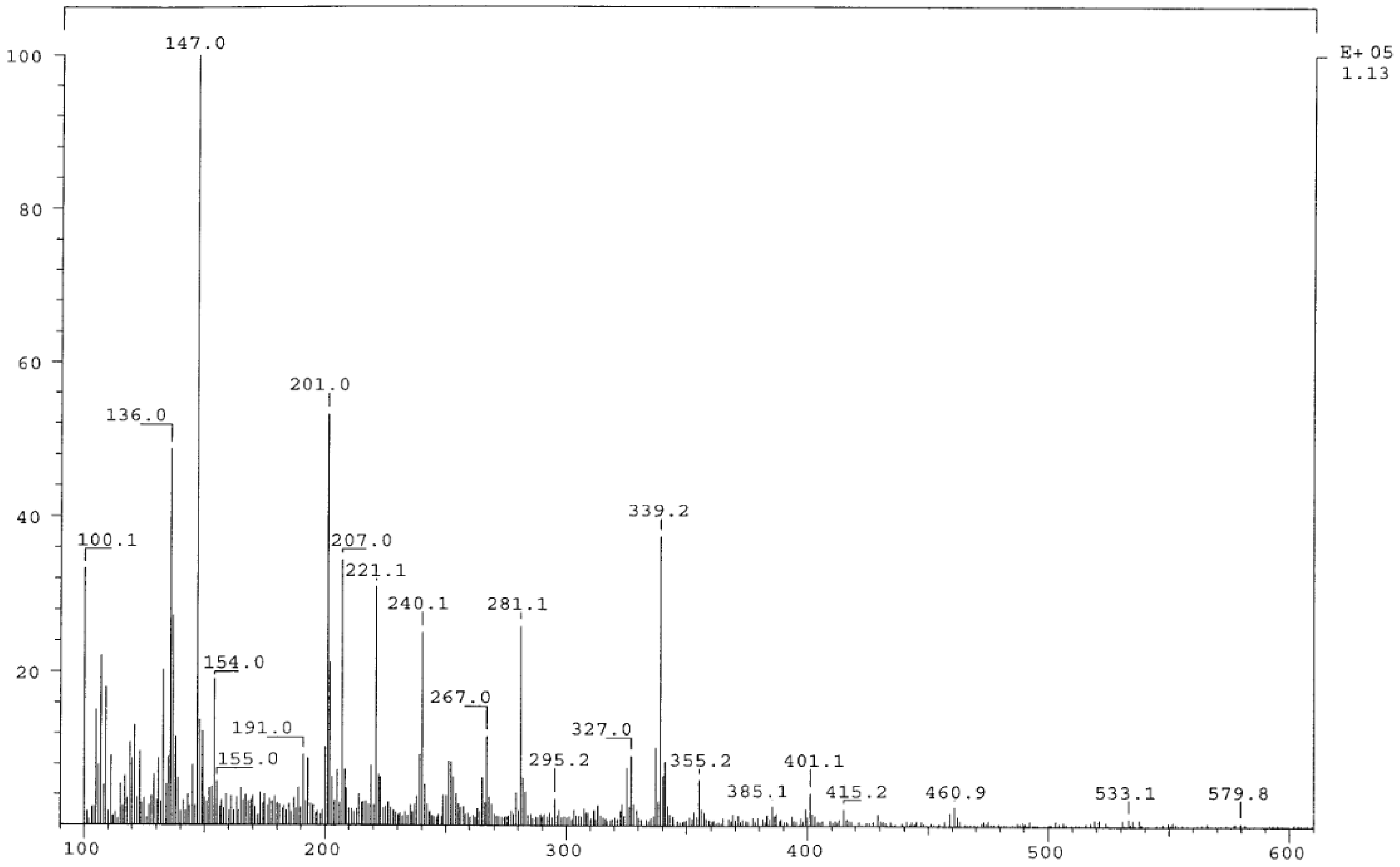

Supplement: Supplementary file 1 [file molecules-25-02604-s001.pdf]
